# Prenatal health behaviours as predictors of human placental lactogen levels

**DOI:** 10.3389/fendo.2022.946539

**Published:** 2022-09-09

**Authors:** Samantha M. Garay, Lorna A. Sumption, Rosalind M. John

**Affiliations:** School of Biosciences, Cardiff University, Cardiff, Wales, United Kingdom

**Keywords:** placental lactogen, birth weight, maternal depression, health-conscious diet, body mass index

## Abstract

Placental lactogen (hPL) is a key hormone of pregnancy responsible for inducing maternal adaptations critical for a successful pregnancy. Low levels of placental lactogen have been associated with lower birth weight as well as symptoms of maternal depression and anxiety. Lower placental lactogen has been reported in women with higher body mass index (BMI) but it is unclear whether prenatal health behaviours predict hPL levels or if hPL is associated with infant weight outcomes. This study utilised data from the longitudinal Grown in Wales cohort, based in South Wales. Participants were recruited at the pre-surgical appointment for an elective caesarean section. This study incorporates data from recruitment, post-delivery and a 12 month follow-up. Measures of maternal serum hPL were available for 248 participants. Analysis included unadjusted and adjusted linear and binary regression. Unadjusted, prenatal smoking and a Health Conscious dietary pattern were associated with hPL levels, however this was lost on adjustment for BMI at booking, Welsh Index of Multiple Deprivation (WIMD) score and placental weight. When stratified by maternal BMI at booking, a Health Conscious dietary pattern remained associated with increased hPL levels in women with a healthy BMI (p=.024, B=.59. 95% CI=.08,1.11) following adjustment for WIMD score and placental weight. When adjusted for a wide range of confounders, maternal hPL was also associated with increased custom birthweight centiles (CBWC) (p=.014, B=1.64. 95% CI=.33,2.94) and increased odds of large for gestational age deliveries (p=<.001, Exp(B)=1.42. 95% CI=1.17,1.72). This study identified that consuming a Health Conscious dietary pattern in pregnancy was associated with increased hPL, within women of a healthy BMI. Moreover, higher hPL levels were associated with increased CBWC and increased odds of delivering a large for gestational age infant. This improves the current limited evidence surrounding the nature of hPL in these areas.

## Introduction

During pregnancy the mammalian mother undergoes substantial adaptations to support fetal development and to prepare for nurturing her offspring once they are born (1–5). Playing an essential role in these maternal adaptations are the hormones produced by, or dependent on, the fetally-derived placenta. Placental hormones ensure sufficient nutrients are available to support fetal and placental growth by increasing maternal appetite, decreasing her activity and driving metabolic adaptations throughout pregnancy (6, 7). Placental hormones are also involved in inducing behavioural changes in the mother during pregnancy priming her to respond expeditiously to her offspring when they are born (8–13). Consequently, constraints in the production of placental hormones can have wide reaching consequences for fetal growth, maternal metabolism and maternal behaviour potentially contributing to the co-morbidity of common complications of pregnancy.

Human placental lactogen (hPL) is one of the key hormones of pregnancy, and the most highly expressed peptide hormone of the human placenta (14). hPL is collectively composed of two identical placental lactogen peptides encoded by *CHORIONIC SOMATOMAMMOTROPIN HORMONE 1* and *2* (aka *HPL-A* and *HPL-B*) (15). Placental lactogens are evolutionarily related to the pituitary hormone prolactin (15) and signal *via* the prolactin receptor to mediate their activity at target sites around the body (15, 16). During pregnancy hPL is synthesised in increasing amounts by the syncytiotrophoblast and extravillous trophoblast lineages of the human placenta with levels reaching 5–7 µg/ml in maternal blood at term, exceeding that of any other peptide hormone (14, 15, 17). While there are rare cases of pregnancy proceeding in the apparent absence of hPL (18), several studies have reported associations between lower than normal levels of hPL and pregnancy complications (19). For example, reduced levels of maternal serum hPL levels and placental *CSH1/2* mRNA expression have been associated with fetal growth restriction (20–22) while positive correlations have been reported between hPL and birthweight (23, 24). One study reported reduced serum hPL in gestational diabetes (25) while another reported significantly reduced placental *CSH1/2* associated with pre-eclampsia (26). We reported an association between lower placental *CSH1/2* at term and both clinically diagnosed depression and questionnaire reported symptoms of depression in pregnancy (27). More recently, we reported low serum placental lactogen at term was associated with symptoms of both depression and anxiety for up to ten weeks after birth (28). In this same study we noted a positive association between serum hPL and birthweight (g), placental weight and head circumference consistent with previous studies. While these studies in human populations do not demonstrate a causal relationship between placental lactogen and birthweight, gestational diabetes or maternal mental health, data from rodent models supports such a conclusion. For example, transgenic overexpression of mouse placental lactogen targeted to the beta cells of the pancreas increases the proliferation rate of these cells in mice, and drives both fasting and postprandial hypoglycaemia (29) while targeted deletion of the prolactin receptor provides indirect evidence that placental lactogens drive pancreatic β-cell expansion (30). Infusion of placental lactogen into the non-pregnant female rodent brain stimulates maternal caregiving behaviour (31, 32) while ablation of the maternal prolactin receptor disrupts maternal caregiving (33–37). Disruption of signalling *via* this receptor has also been linked to increased postpartum anxiety (38). Our work on mice with placental endocrine insufficiency driven by genetically modified changes in the expression of imprinted genes further demonstrates a role for placental hormones in regulating birthweight with a reduction in the number of placental endocrine cells linked to low birthweight in several models (39–41). We also reported both maternal neglect and maternal anxiety in response to the loss of placental endocrine lineages (42, 43) with the mouse offspring exhibiting anxiety-like behaviours later in life (44). Together, these data highlight the importance of placental hormones, and more specifically placental lactogens, for pregnancy health. Moreover, in addition to genetic drivers of placental endocrine insufficiency, a number of environmental stressors in pregnancy have been linked to changes in the expression of placental hormones and alterations in maternal behaviour (45) identifying a mechanism with potential to link early life adversity to a variety of poor health outcomes.

Given the importance of placental lactogen for a healthy and successful pregnancy, it is vital that we identify factors that positively or negatively influence the production of this hormone. Previously pre-pregnancy obesity has been linked to significantly lower placental expression of *CSH1/2* (46–48). Similarly, we have reported an association between maternal BMI at booking (week 12-14 of pregnancy) and serum hPL at term (28). In addition, we noted an association between serum hPL and the Welsh Index of Multiple Deprivation (WIMD) score. WIMD is the Welsh Government’s official measure of relative deprivation for small areas in Wales calculated from anonymised postcodes (http://wimd.wales.gov.uk). The small areas used to construct the index are known as Lower Super Output Areas (LSOAs) with an average population of 1,600 people. There are 1,909 LSOAs in Wales - the most deprived area is given a rank of 1 and the least deprived a rank of 1,909 therefore lower scores are indicative of higher levels of deprivation. The WIMD is composed of a number of indicators which include income, employment, health, education, access to services, housing, community safety and physical environment. We have previously reported that WIMD scores were significantly positively associated with a ‘Health Conscious’ dietary pattern which in turn was significantly associated with increased custom birthweight centile (CBWC) (49). Together, these observations suggest that factors in addition to maternal BMI may influence hPL levels. Here we explored the association between a variety of modifiable health behaviours in pregnancy and term serum hPL, as well as the influence of hPL on a range of infant weight outcomes, using data from the Grown in Wales Study.

## Material and methods

### Cohort

This study analysed data from the Grown in Wales (GiW) cohort, a pregnancy cohort recruited in South Wales, UK with a focus on maternal mental health (50). Full ethical approval for the GiW study was obtained by the Wales Research Ethics Committee (REC2 reference 15/WA/0004). Research was carried out employing the principles of the Declaration of Helsinki as revised in 2008. Recruitment occurred between September 2015 and November 2016 at the University Hospital of Wales (UHW) with women providing written consent to the study. Women were recruited by two trained research midwives at their morning pre-surgical appointment in advance of an elective caesarean (ELCS), one to four days before delivery. ELCS was chosen to maximise the potential for collecting biological samples. At UHW women routinely provide blood samples at their pre-surgical appointment before their surgery facilitating the collection of maternal blood for this study. The planned surgery took place during the working week when the research midwives were available to collect placental biopsies and cord blood samples. Recruitment criteria consisted of women being between 37 weeks and 42 weeks of pregnancy, aged between 18 and 45, having a singleton birth without fetal abnormalities or infectious diseases. Participants have been followed up within one week of birth, ten weeks and one year postnatally and most recently at four years postpartum.

### Participants

355 women were initially recruited and seven later withdrew. Of these, hPL measures were available 272 participants within the overall cohort. The current analysis focused on participants who delivered at term (≥ 37 weeks) and those of Caucasian ethnicity. This selection was required as the dietary patterns were previously developed for these participants (49). This was due to the recruitment small number of participants of other ethnicities whose inclusion greatly influenced findings through the introduction of variation, an issue especially relevant for smaller cohorts (51, 52). Following the exclusion criteria, hPL data was available for 248 participants.

### Human placental lactogen

Maternal venous serum samples were obtained at recruitment from blood taken as part of a standard anaesthetic review one to four days prior to surgery. Serum was obtained by centrifugation of maternal venous blood which was then frozen at −80°C. hPL levels were assayed in duplicate using the Leinco Technologies Human Placental Lactogen (HPL) Micro-ELISA test kit (Universal Biologicals product code T115-96 tests). Assays were performed by the NIHR Cambridge Biomedical Research Centre, Core Biochemical Assay Laboratory. Average value = 8.3 µg/mL ± 2.75.

### Demographic and biological data

Demographic data such as a participants education level and income were obtained from the maternal questionnaire completed at recruitment. Participant postcodes were also collected and anonymised which enabled the calculation of Welsh Index of Multiple Deprivation (WIMD) 2014 scores (http://wimd.wales.gov.uk). The maternal questionnaires also contained the Edinburgh Postnatal Depression Scale (EPDS) (53) and the State-Trait Anxiety Inventory (STAI) (54) which provided data on maternal mental health. Biological data such as participants ethnicity, age, parity, weight and BMI at booking as well as data on mode of delivery, placental weight and infant sex were collected from the midwife recorded notes following delivery. Gestational weight gain was calculated from data on pre-pregnancy weight and weight at booking.

### Prenatal health behaviours

Data on maternal prenatal smoking, alcohol intake and exercise were acquired from the maternal questionnaire completed at recruitment. Dietary patterns were identified from data collected through a food frequency questionnaire (FFQ), also completed at recruitment. The dietary patterns within the GiW cohort were Western and Health Conscious, with the process for obtaining the dietary patterns outlined in detail in (49). Briefly, the dietary pattern scores were obtained *via* the regression method following Principal Component Analysis. Each participant has a score for both dietary patterns. These scores are typically centred around zero, with greater positive scores indicating higher adherence to a dietary pattern and greater negative scores indicating lower adherence to a dietary pattern.

### Infant weight outcomes

Data on birthweight (g) was obtained from the midwife recorded notes following delivery. CBWC were later calculated *via* the GROW bulk centile calculator (55) utilising the following data from the midwife notes; maternal height, weight, ethnicity and parity as well as infant gender, birthweight and gestational age. This enabled the classification of infant birthweight as small for gestational age (SGA), average for gestational age (AGA) or large for gestational age (LGA). Data on infant weight at one year of age was obtained from a maternally completed questionnaire.

### Statistical analysis

All statistical analyses were undertaken utilising IBM SPSS Statistics Version 27. Normality for relevant variables was assessed *via* Kolmogorov-Smirnov test, Shapiro-Wilk test, normal Q-Q plots and histograms. All relevant variables were determined to be non-parametric, thus demographic statistics were displayed as median (IQR) or % (n) as appropriate. Health behaviour predictors of hPL were assessed utilising both unadjusted and adjusted linear regression. In the adjusted analysis the significant predictors were entered together in the model, with maternal BMI at booking (continuous), WIMD score and placental weight (g) selected as potentially confounding variables. Variables were selected as confounding variables if a previous GiW study identified them to be associated with hPL (28) or if the variables were found to be associated with the outcome variables in a univariate analysis (**Supplementary Table 1**). In light of the highly influential nature of maternal BMI, the association between predictors and hPL was also assessed when stratified by maternal BMI at booking, with the exception of the underweight BMI category due to low numbers. Unadjusted and adjusted linear and binary regression were undertaken to assess the influence of hPL on infant weight outcomes. The analysis of birthweight (g) was adjusted for the following potentially confounding variables; BMI at booking (continuous), WIMD score, maternal age, gestational weight gain fetal sex, placental weight (g), gestational age, smoking and a Health Conscious dietary pattern. Education and income were not included as the WIMD score incorporates these measures. The same variables were utilised for analyses of CBWC derived variables, with the exception of fetal sex and gestational age which are already accounted for within this birthweight measure.

## Results

Demographic data for the 248 participants involved in the analysis is provided in **Table 1**. Categorical data is displayed as % (n) and continuous data as median (IQR).

**Table 1:** Demographic data for the eligible GiW participants.

	% (n) or median (IQR)
Maternal BMI at booking - overall	26.33 (7.23)
Maternal BMI at booking % (n)	
Underweight	.40 (1)
Healthy	38.20 (89)
Overweight	35.60 (83)
Obese	25.80 (60)
Maternal age at booking	33.00 (6.00)
Parity, *% (n)*	
Multiparous	81.90 (203)
Nulliparous	18.10 (45)
Gestational weight gain (kg)	15.07 (7.88)
GDM % (n)	
Yes	5.30 (13)
No	94.70 (230)
Hypertension % (n)	
Yes	3.70 (9)
No	96.30 (236)
Fetal sex, *% (n)*	
Female	54.40 (135)
Male	45.60 (113)
Placental weight (g)	655 (183)
Gestational age (weeks)	39.00 (0)
Birthweight (g)	3500.00 (650.00)
Birthweight classification	
LBW	2.60 (8)
ABW	79.20 (247)
HBW	18.30 (57)
CBWC	57.85 (50.05)
Size for gestational age *% (n)*	
SGA	6.90 (17)
AGA	80.60 (200)
LGA	12.50 (31)
Smoking in pregnancy^a^, *% (n)*	
No	89.80 (220)
Yes	10.20 (25)
Alcohol in pregnancy^a^, *% (n)*	
No	59.30 (144)
Yes	40.70 (99)
Strenuous exercise, *% (n)*	
No	81.60 (200)
Yes	18.40 (45)
Western dietary pattern	-.03 (1.28)
Health Conscious dietary pattern	.05 (1.50)
Highest education level, *% (n)*	
Left before GCSE	5.90 (14)
GCSE & Vocational	22.90 (54)
A-level	12.70 (30)
University	30.90 (73)
Postgraduate	27.50 (65)
Family income (£), *% (n)*	
<18,000	7.50 (18)
18 – 25,000	10.00 (24)
25-43,000	19.70 (47)
>43,000	52.30 (125)
Do not wish to say	10.50 (25)
WIMD	1270.00 (1211.00)
A1 EPDS total	7.00 (6.00)
A1 STAI total	34.00 (13.00)

IQR, Interquartile range; BMI, body mass index; GDM, gestational diabetes mellitus; LBW, low birthweight; ABW, average birthweight; HBW, high birthweight; CBWC, custom birthweight centile; SGA, small for gestational age; AGA, average for gestational age; LGA, large for gestational age; WIMD, Welsh Index of Multiple Deprivation; EPDS, Edinburgh Postnatal Depression Scale; STAI, State-Trait Anxiety Inventory.

a At any point in pregnancy.

### Health behaviour predictors of hPL

Linear regression was utilised to investigate if prenatal maternal health behaviours influence levels of maternal serum hPL (**Table 2**). Unadjusted univariate regression identified that both smoking at any point in pregnancy and a Health Conscious dietary pattern were associated with hPL measures. Specifically, smoking compared to not smoking was associated with a decrease in hPL of 1.24 µg/mL, whilst a one unit increase in Health Conscious dietary pattern score was associated with an increase in hPL of.40 µg/mL. These significant health behaviours were adjusted for: maternal BMI at booking, WIMD score and placental weight (g). Following adjustment, no prenatal health behaviours remained significantly associated with hPL measures. To understand the highly influential effect of BMI further, the relationship between health behaviours and hPL was examined when stratified by maternal BMI at booking (**Table 3**). It was determined that a Health Conscious dietary pattern was significantly associated with hPL measures in women classified as having a healthy BMI at booking. This association remained after adjustment for WIMD score and placental weight (g). Specifically, for women with a healthy BMI at booking, a one unit increase in Health Conscious dietary pattern score was associated with an increase in hPL of .59 µg/mL equivalent to an increase of 8% of the average value.

**Table 2:** Unadjusted and adjusted linear regression indicating the association between maternal prenatal health behaviours and hPL (μg/mL).

		*p*	B	95% CI
Unadjusted	Smoking	**.036**	-1.24	-2.40, -.08
	Alcohol	.359	.33	-.38, 1.04
	Exercise	.119	.72	-.19, 1.63
	Western dietary pattern	.640	-.09	-.47, .29
	Health Conscious dietary pattern	**.029**	.40	.04, .76
Adjusted	Smoking	.706	-.23	-1.42, .97
	Health Conscious dietary pattern	.618	.09	-.26, .44

CI, confidence interval. Bold values are significant at p < .05.

**Table 3:** Unadjusted and adjusted linear regression indicating the association between maternal prenatal health behaviours and hPL (μg/mL) when stratified by maternal BMI at booking.

			*p*	B	95% CI
Unadjusted	Healthy	Smoking	.179	-1.43	-3.53, .67
		Alcohol	.259	.66	-.49, 1.81
		Exercise	.264	.72	-.56, 2.00
		Western dietary pattern	.227	-.36	-.95, .23
		Health Conscious dietary pattern	**.020**	.65	.11, 1.19
	Overweight	Smoking	.321	-1.29	-3.85, 1.28
		Alcohol	-1.14	.23	-1.14, 1.61
		Exercise	.837	.22	-1.93, 2.38
		Western dietary pattern	.720	-.14	-.88, .61
		Health Conscious dietary pattern	.338	.35	-.37, 1.07
	Obese	Smoking	.448	-.66	-2.40, 1.08
		Alcohol	.914	.07	-1.28, 1.43
		Exercise	.161	1.39	-.57, 3.34
		Western dietary pattern	.214	.45	-.27, 1.17
		Health Conscious dietary pattern	.404	-.33	-1.10, .45
Adjusted	Healthy	Health Conscious dietary pattern	**.024**	.59	.08, 1.11

CI, confidence interval. Bold values are significant at p < .05.

### hPL & infant weight outcomes

**Figure 1:**
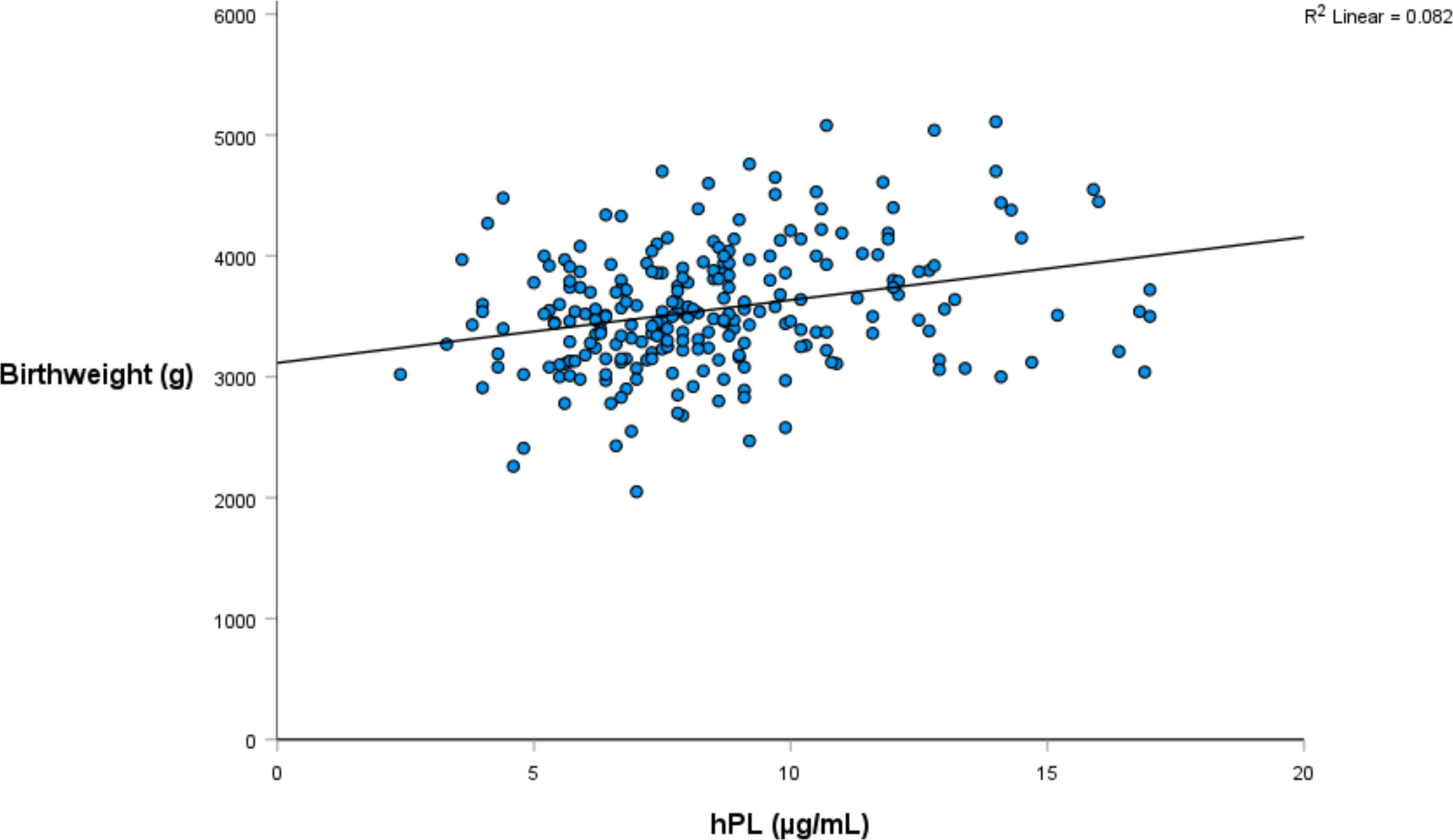
The relationship between hPL (µg/mL) and infant birthweight (g).

**Figure 2:**
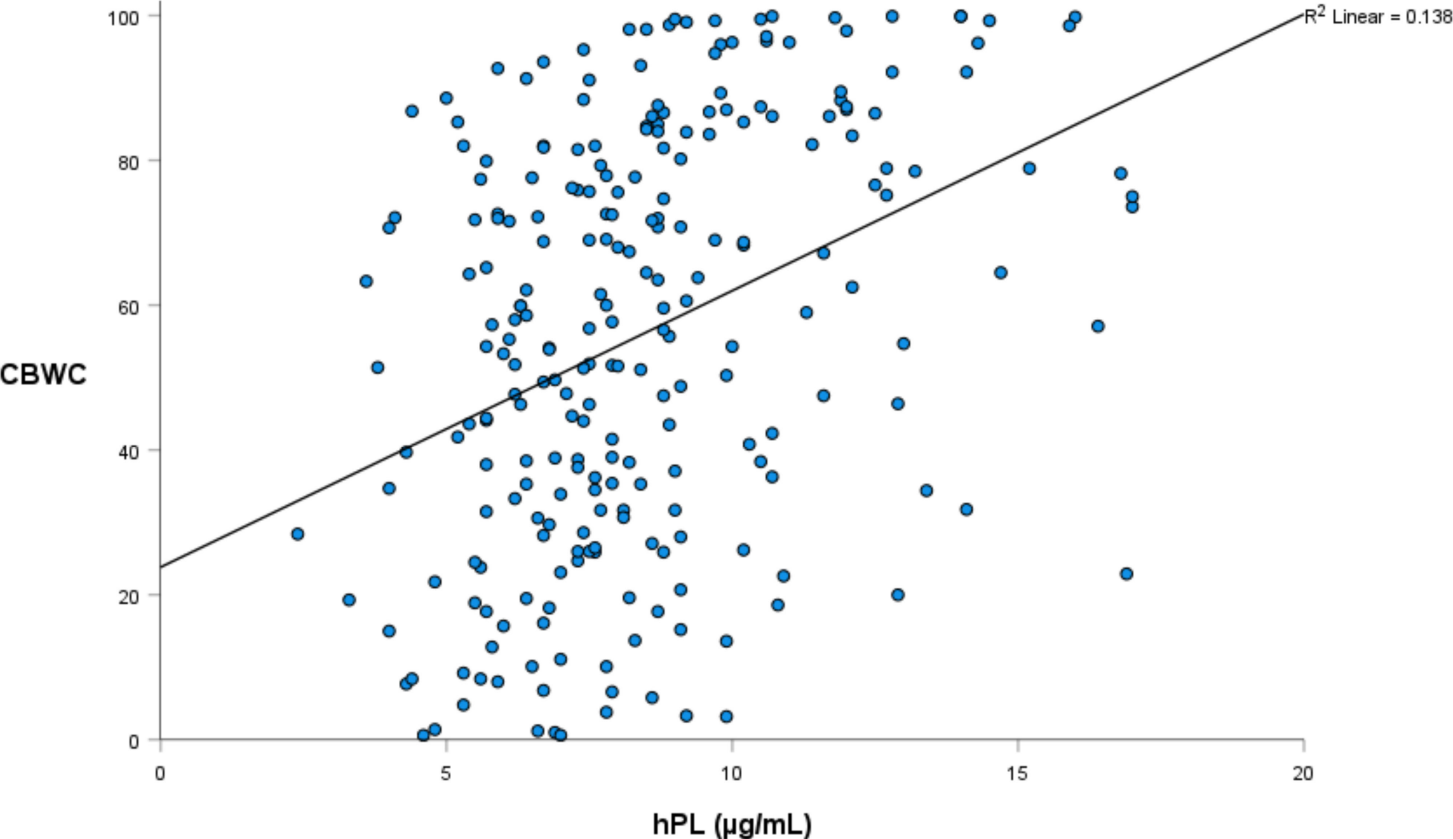
The relationship between hPL (µg/mL) and infant custom birthweight centile.

Linear regression was again utilised to investigate the relationship between hPL and a range of infant weight measures, collected both at birth and at one year of age (**Tables 4**, **5**). The relationship between hPL and both birthweight and CBWC is displayed in **Figures 1**, **2**. At the unadjusted level, hPL was significantly associated with all measures of weight with the exception of infant weight at 12 months of age. These significant associations were adjusted for the potentially confounding variables that included maternal BMI at booking, maternal age, gestational weight gain, fetal sex, placental weight, gestational age, smoking at any point in pregnancy, Health Conscious dietary pattern score and WIMD score. Following adjustment, hPL was no longer significantly associated with birthweight (g) or the odds of being born SGA compared to LGA. However, hPL remained significantly associated with CBWC, with a one unit increase in hPL associated with an increase in CBWC of 1.64 units. Additionally, hPL was associated with being born LGA compared to AGA, with a one unit increase in hPL associated with increased odds of delivering an LGA compared to AGA infant by a factor of 1.42.

**Table 4:** Unadjusted and adjusted linear regression indicating the association between hPL (μg/mL) and infant weight outcomes.

		*p*	B	95% CI
Unadjusted	Birthweight (g)	**<.001**	52.01	30.20, 73.82
	CBWC	**<.001**	3.82	2.62, 5.01
	12 month weight (kg)	.967	.00	-.17, .17
Adjusted	Birthweight (g)	.090	16.56	-2.64, 35.75
	CBWC	**.014**	1.64	.33, 2.94

CI, confidence interval; CBWC, custom birthweight centile. Bold values are significant at p < .05.

**Table 5:** Unadjusted and adjusted binary regression indicating the association between hPL (μg/mL) and infant weight categories.

		*p*	Exp (B)	95% CI
Unadjusted	SGA	**.008**	.70	.53, .91
	LGA	**<.001**	1.31	1.15, 1.49
Adjusted	SGA	.090	.72	.49, 1.05
	LGA	**<.001**	1.42	1.17, 1.72

CI, confidence interval; SGA, small for gestational age; LGA, large for gestational age. Bold values are significant at p < .05.

## Discussion

This study aimed to investigate both the health behaviour predictors of hPL and the influence of hPL on infant weight outcomes. It was determined that, at the unadjusted level, both smoking at any point in pregnancy and consuming a Health Conscious dietary pattern were associated with hPL levels. However, this was lost following adjustment for the confounding variables of WIMD score, maternal BMI at booking and placental weight. Given that BMI is known to strongly influence maternal hPL, this association was also examined when stratified by BMI. Following adjustment for WIMD score and placental weight, consuming a Health Conscious dietary pattern in pregnancy was associated with increased hPL levels in participants with a healthy BMI at booking. Regarding infant weight outcomes, prior to adjustment hPL was associated with all weight outcomes with the exception of infant weight at 12 months. This analysis was adjusted for a range of confounding variables including maternal BMI at booking, maternal age, gestational weight gain, fetal sex, placental weight, gestational age, smoking at any point in pregnancy, Health Conscious dietary pattern score and WIMD score. After adjustment, maternal hPL was associated with increased CBWC as well as increased odds of delivering an LGA compared to AGA infant.

The associations between the modifiable prenatal health behaviours of maternal smoking and adhering to a Health Conscious diet on hPL were identified but did not remain associated once adjusted for BMI at booking, WIMD score and placental weight. However, given that BMI is known to be highly influential for hPL levels, this analysis was stratified by BMI at booking. When stratified, the association between a Health Conscious dietary pattern and hPL remained significant for women within the healthy BMI category. This finding supports the important influence of maternal diet in relation to hPL levels. Moreover, there is potential for this to be a direct relationship with studies in several experimental animal models reporting that both overnutrition and undernutrition reduce the expression of placental hormones (45). However, while this relationship was evident in women of a healthy BMI, it was not apparent in women with an unhealthy BMI. BMI is already known to have an influential effect on maternal hPL serum levels with structural changes in the placental *hPL* gene locus reported in women with higher BMI compared to those on the normal range (48). Together, these findings suggest that BMI has a stronger influence on hPL levels than maternal diet. The caveat is that, in our cohort, BMI and diet are linked with increasing BMI associated with decreasing Health Conscious dietary pattern score (49). Similarly in the majority of animal models of overnutrition, both weight gain and exposure to diet occur concurrently in pregnancy. Distinguishing direct and indirect relationships consequently presents a challenge.

We have previously reported a positive relationship between term serum hPL and infant birthweight (g), head circumference and placental weight (g) (28) consistent with a number of previous studies (20, 21). This study went further by examining additional weight measures. CBWC and the associated classifications of SGA, AGA and LGA have several advantages over the traditional population based weight measures (55, 56) and have been recommended for use in the UK by the Royal College of Obstetricians and Gynaecologists since 2002 (57). In our cohort nearly twice the number of infants would be classified as growth restricted by the CBWC criteria (**Table 1**). However, these measures are rarely utilised in research. We are unaware of any other studies reporting the influence of hPL on these birthweight measures. As such, this research strengthens and supports the current evidence base that hPL is associated with birthweight outcomes. As there was no association between hPL and infant weight at 12 months, this also suggests that the influence of hPL on infant weight is short term in nature.

There are several potential limitations to consider regarding this study. Firstly, dietary patterns were originally identified using data from Caucasian participants, a demographic which forms the majority of the Grown in Wales study cohort (91%). As such, the generalisability of the study to other ethnicities may be limited and future research should be conducted with diverse populations to validate the findings. Secondly, our population were recruited to explore the impact of maternal depression on the placenta and therefore focused on recruiting women booked for ELCS. This selective process is both a limitation – due to the restricted nature of the cohort – and an advantage since hPL measures were all taken 1-4 days prior to birth by the same two research midwives on the morning of the participants surgical assessment. This focused timing in the collection of samples and the somewhat homogenous nature of the cohort means that we are able to detect subtle relationships in our relatively small pregnancy cohort. However, an important question remains unanswered which is the timings of the relationships. We have a single measure of hPL at near term. Determining a more precise timeline will be important.

In conclusion, we have established that there is a positive association between a healthy maternal diet and hPL, a key hormone of pregnancy, at least within women with a healthy BMI category. Moreover, hPL is associated with birthweight outcomes. While we have not established the extent to which this is a direct relationship, it is clear that consuming a healthy diet in pregnancy reduces the risk of a number of complications of pregnancy and is likely to protect offspring from the longer term problem association with exposure to early life adversity.

## Data availability statement

The raw data supporting the conclusions of this article will be made available by the authors, without undue reservation, upon request.

## Ethics statement

The studies involving human participants were reviewed and approved by Wales Research Ethics Committee REC2 reference 15/WA/0004. The patients/participants provided their written informed consent to participate in this study.

## Author contribution

RJ: conceptualisation, funding acquisition, project administration, resources, writing – original draft, supervision. SG: data curation, formal analysis, investigation, writing – original draft. LS: data curation, writing – review & editing. All authors contributed to the article and approved the submitted version.

## Acknowledgments

The authors would like to thank participants of the Grown in Wales study, the research midwives Nicola Savory and Anouk Ridgway and past members of the research group including Anna Janssen who contributed to the present study. RJ takes full responsibility for the work as a whole, including the study design, access to data and the decision to submit and publish the manuscript. The Grown in Wales study was funded by MRC grant (MR/M013960/1); SG was supported by a GW4 BioMed MRC DTP PhD studentship (MR/N013794/1) and The Waterloo Foundation (Reference 1403-4488). LS was supported by GW4 SWBio BBSRC DTP PhD studentship (BB/M009122/1). Study design: RJ. Data analysis: LS and SG. Manuscript: RJ and SG. All authors have approved the final article.

## Conflict of interest

The authors declare that the research was conducted in the absence of any commercial or financial relationships that could be construed as a potential conflict of interest.

## Publisher’s note

All claims expressed in this article are solely those of the authors and do not necessarily represent those of their affiliated organizations, or those of the publisher, the editors and the reviewers. Any product that may be evaluated in this article, or claim that may be made by its manufacturer, is not guaranteed or endorsed by the publisher.
