# Supplementary material for: Prenatal health behaviours as predictors of human placental lactogen levels
Source: Front Endocrinol (Lausanne). 2022 Sep 9;13:946539. doi: 10.3389/fendo.2022.946539 (PMC9500170; doi:10.3389/fendo.2022.946539)
Supplement: Supplementary file 1 [file Table_1.docx]

**Table S1. Univariate linear regression assessing the association between collected variables and both maternal hPL (μg/ml) and infant birthweight (g).**

|  | hPL | | | Birthweight (g) | | |
| --- | --- | --- | --- | --- | --- | --- |
|  | p | B | 95% CI | p | B | 95% CI |
| Maternal BMI at booking - overall | **.004** | -.10 | -.17, -.03 | **<.001** | 21.93 | 11.51, 32.36 |
| Maternal BMI at booking |  |  |  |  |  |  |
| Underweight | .852 | -.53 | -6.07, 5.02 | .092 | -28.46 | -61.60, 4.68 |
| Healthy | *ref* |  |  | *ref* |  |  |
| Overweight | .391 | -.37 | -1.21, .48 | .112 | 6.26 | -1.46, 13.97 |
| Obese | **.009** | -1.23 | -2.15, -.31 | .353 | 3.98 | -4.45, 12.42 |
| Maternal age at booking | .620 | .02 | -.05, .09 | **.014** | 14.16 | 2.88, 25.45 |
| Parity |  |  |  |  |  |  |
| Multiparous | *ref* |  |  | *ref* |  |  |
| Nulliparous | .483 | .32 | -.58, 1.23 | .375 | -3.64 | -11.70, 4.42 |
| Gestational weight gain (kg) | .873 | .00 | -.05, .04 | .**019** | 8.70 | 1.47, 15.93 |
| GDM |  |  |  |  |  |  |
| Yes | .475 | .57 | -1.00, 2.13 | .102 | 232.20 | -46.36, 510.76 |
| No | *ref* |  |  | *ref* |  |  |
| Hypertension |  |  |  |  |  |  |
| Yes | .672 | -.40 | -2.27, 1.46 | .379 | -153.56 | -496.48, 189.37 |
| No | *ref* |  |  | *ref* |  |  |
| Fetal sex, *% (n)* |  |  |  |  |  |  |
| Female | *ref* |  |  | *ref* |  |  |
| Male | .467 | -.26 | -.96, .44 | **<.001** | 267.39 | 155.63, 379.15 |
| Placental weight (g) | **<.001** | .01 | .01, .01 | **<.001** | 2.39 | 2.09, 2.70 |
| Gestational age (weeks) | .656 | .12 | .66, -.41 | **<.001** | 212.55 | 133.88, 291.23 |
| Smoking in pregnancy^a^ | - | - | - |  |  |  |
| No |  |  |  | *ref* |  |  |
| Yes |  |  |  | **.001** | -306.89 | -488.79, -125.00 |
| Alcohol in pregnancy^a^ | - | - | - |  |  |  |
| No |  |  |  | *ref* |  |  |
| Yes |  |  |  | .869 | 10.07 | -110.14, 130.27 |
| Strenuous exercise | - | - | - |  |  |  |
| No |  |  |  | *ref* |  |  |
| Yes |  |  |  | .337 | -75.79 | -230.75, 79.16 |
| Western dietary pattern | - | - | - | .188 | -38.86 | -96.84, 19.13 |
| Health Conscious dietary pattern | - | - | - | **<.001** | 102.94 | 45.97, 159.91 |
| Highest education level |  |  |  |  |  |  |
| Left before GCSE | .072 | -1.47 | -3.07, .13 | **.006** | -351.14 | -602.66, -99.62 |
| GCSE & Vocational | .583 | -.28 | -1.26, .71 | .066 | -146.95 | -303.52, 9.63 |
| A-level | .727 | .21 | -.98, 1.40 | .423 | 77.95 | -113.17, 269.08 |
| University | *ref* |  |  | *ref* |  |  |
| Postgraduate | .222 | .58 | -.35, 1.52 | .927 | -7.18 | -162.08, 147.71 |
| Family income (£) |  |  |  |  |  |  |
| <18,000 | .262 | -.78 | -2.16, .59 | .**017** | -257.19 | -468.82, -46.56 |
| 18 – 25,000 | .071 | -1.12 | -2.33, .10 | .859 | -18.43 | -222.55, 185.70 |
| 25-43,000 | .131 | -.72 | -1.65, .21 | **.037** | -160.40 | -311.04, -9.76 |
| >43,000 | *ref* |  |  | *ref* |  |  |
| Do not wish to say | .920 | .06 | -1.13, 1.25 | .112 | -148.94 | -332.98, 35.10 |
| WIMD | .**009** | .00 | .00, .00 | **.031** | .10 | .01, .19 |
| A1 EPDS total | .086 | -.07 | -.14, .01 | .660 | 2.83 | -9.74, 15.51 |
| A1 STAI total | .171 | -.03 | -.07, .01 | .503 | 2.41 | -4.67, 9.50 |
